# Powdery mildew resistance of barley accessions from Dagestan

**DOI:** 10.18699/VJ21.059

**Published:** 2021-09

**Authors:** R.A. Abdullaev, T.V. Lebedeva, N.V. Alpatieva, B.A. Batasheva, I.N. Anisimova, E.E. Radchenko

**Affiliations:** Federal Research Center the N.I. Vavilov All-Russian Institute of Plant Genetic Resources (VIR), St. Petersburg, Russia; Federal Research Center the N.I. Vavilov All-Russian Institute of Plant Genetic Resources (VIR), St. Petersburg, Russia; Federal Research Center the N.I. Vavilov All-Russian Institute of Plant Genetic Resources (VIR), St. Petersburg, Russia; Dagestan Experiment Station of VIR, N.I. Vavilov All-Russian Institute of Plant Genetic Resources (VIR), Vavilovo Village, Derbent, Dagestan, Russia; Federal Research Center the N.I. Vavilov All-Russian Institute of Plant Genetic Resources (VIR), St. Petersburg, Russia; Federal Research Center the N.I. Vavilov All-Russian Institute of Plant Genetic Resources (VIR), St. Petersburg, Russia

**Keywords:** barley landraces, *Blumeria graminis* f. sp. *hordei*, durable resistance, genes, образцы местного ячменя, *Blumeria graminis* f. sp. *hordei*, длительная устойчивость, гены

## Abstract

Powdery mildew caused by the parasitic fungus *Blumeria graminis* (DC.) Golovin ex Speer f. sp. *hordei* Marchal is one of the most common diseases of barley. Growing resistant varieties can signif icantly minimize harmful effects of the pathogen. The specif icity in the interaction between the fungus and its host plant requires a continuous search for new donors of the resistance trait. The powdery mildew resistance of 264 barley accessions from Dagestan and genetic control of the trait in resistant forms were studied under f ield and laboratory conditions. Forty-seven barley lines carrying previously identif ied powdery mildew resistance genes were also examined. During three years, the experimental material was evaluated under severe infection pressure at the Dagestan Experiment Station of VIR (North Caucasus, Derbent). Juvenile resistance against the Northwest (St. Petersburg, Pushkin) pathogen population was evaluated in a climatic chamber. The genetic control of *B. graminis* resistance in the selected accessions was studied with the application of hybridological and molecular analyses. The level of genetic diversity of Dagestan barley for effective resistance to powdery mildew is very low. Only two accessions, VIR-23787 and VIR-28212, are resistant against *B. graminis* at both seedling and adult plant stages. The high-level resistance of breeding line VIR-28212 originating from barley landrace VIR-17554 (Ep-80 Abyssinien) from Ethiopia is controlled by the recessive gene *mlo11*. Accession VIR-17554 is heterogeneous for the studied trait, with the powdery mildew resistant genotypes belonging to two varieties, *dupliatrum* (an awnless phenotype) and *nigrinudum* (an awned phenotype). In accession VIR-23787, a recessive resistance gene distinct from the *mlo11* allele was identif ied. This accession is supposed to be protected by a new, effective pathogen resistance gene.

## Abstract (ru)

Мучнистая роса (возбудитель *Blumeria graminis* (DC.) Golovin ex Speer f. sp. *hordei* Marchal) – одно из самых распространенных заболеваний ячменя. Возделывание устойчивых сортов может существенно ограничить вредоносность патогена. Специфичность взаимодействия гриба с растением-хозяином обусловливает необходимость постоянного поиска новых доноров устойчивости. Носителями эффективных генов устойчивости к патогену часто являются образцы местного ячменя. В полевых и лабораторных экспериментах оценили потенциал изменчивости 264 образцов ячменя из Дагестана по устойчивости к *B. graminis* и изучили генетический контроль признака у выделившихся форм. Исследовали также 47 линий ячменя, несущих ранее идентифицированные гены устойчивости к мучнистой росе. В течение трех лет с использованием балловой шкалы экспериментальный материал оценивали на жестком инфекционном фоне в условиях Дагестанской опытной станции Всероссийского института генетических ресурсов растений им. Н.И. Вавилова (Северный Кавказ, г. Дербент), а в климатической камере исследовали ювенильную устойчивость растений к северо-западной (Санкт-Петербург, г. Пушкин) популяции гриба. Генетический контроль устойчивости к *B. graminis* у выделенных образцов изучали с помощью гибридологического и молекулярного анализов. Установили, что генетическое разнообразие ячменей Дагестана по эффективной устойчивости к мучнистой росе весьма невелико. Лишь образцы к-23787 и к-28212 устойчивы к дагестанской популяции *B. graminis* в фазе цветения и к петербургской популяции патогена – в фазе двух листьев. Две географически очень разобщенные популяции гриба оказались идентичны по вирулентности к линиям с ранее идентифицированными генами устойчивости к мучнистой росе. Высокая устойчивость селекционной линии к-28212, полученной с использованием образца местного ячменя из Эфиопии к-17554 (Ep-80 Abyssinien), контролируется рецессивным геном *mlo11*. Образец к-17554 гетерогенен по изученному признаку, при этом устойчивые к мучнистой росе генотипы относятся к двум разновидностям: *dupliatrum* (остистый фенотип) и *nigrinudum* (безостый фенотип). У образца к-23787 обнаружен рецессивный ген устойчивости, отличающийся от аллеля *mlo11*. Предполагается, что этот образец защищен новым эффективным геном устойчивости к патогену.

## Introduction

Powdery mildew caused by the parasitic fungus *Blumeria graminis* (DC.) Golovin ex Speer f. sp. *hordei* Marchal) is one of the most common and harmful diseases of barley. The pathogen predominantly affects leaves, leaf sheath, and stems throughout the growing season. In the infected plants, photosynthetic activity of leaves is being decreased while water loss and respiration intensity are being increased, which results in growth retardation, reduced tillering ability, and decreasing seed mass and grain number per spike. Yield reduction caused by powdery mildew can reach 30 %, with an average of 5–10 % across all regions (Balkema-Boomstra, Masterbroek, 1995; Gong et al., 2013).

Selection of resistant plant genotypes is a radical and environment friendly way to combat the disease. Unfortunately, the pathogen is characterized by differential interaction with the host plant genotype. This means that the ubiquitously observed genetic uniformity of cultivated varieties creates conditions for adaptive microevolution of the fungus.

In barley, numerous powdery mildew resistance genes designated by various symbols have been identified, most of them are alleles at the loci *Mla* and *Mlo*. Thus, 39 alleles at the *Mla (Mildew resistance locus a)* (chromosome 1H) and 44 alleles at the *Mlo (Mildew resistance locus o)* locus (chromosome 4H) are known (Jørgensen, 1994; Seeholzer, 2009; Reinstädler et al., 2010; Kusch, Panstruga, 2017). However, most alleles are ineffective against the causative agent. The allele *mlo11* is practically the only effective gene that confers durable resistance to the pathogen. Barley landraces often possess effective genes for resistance against phytopathogens. For example, a study of 925 Ethiopian barley accessions has revealed 15 accessions harboring the *mlo11* allele, and 59 forms whose resistance to *B. graminis* was controlled by effective genes distinct from the mlo11 (Abdullaev et al., 2019).

Since recently, the attention of researchers is drawn to Dagestan, a region of ancient agriculture. In a small area, very contrasting soil, climatic and landscape conditions are present: from the low-lying Caspian basins to high mountains with eternal snows, from the semi-desert and desert sharply continental northern dry-steppe zones to the regions of the subtropical type. The results of the study of US Barley Collection (USDA National Small Grains Collection) accessions for resistance against several pathogens and insects have shown that the Caucasus could be considered as the “center of concentration” of yellow rust resistant barley forms (the causative agent Puccinia striiformis Westend. f. sp. hordei). In these studies, three accessions from Dagestan were highly resistant to yellow rust and net blotch (Pyrenophora teres (Died.) Drechsl.), as well as moderately resistant to spot blotch (Cochliobolus sativus (Ito et Kurib.) Drechsler ex Dastur.) and viral diseases (Bonman et al., 2005). Powdery mildew resistance of Dagestan barleys has not yet been studied.

This study is aimed at evaluating variability potential of barley accessions from Dagestan for resistance to *B. graminis*, and at elucidating genetic control of the trait in the selected resistant forms.

## Materials and methods

The material used in the study included 264 barley accessions (187 spring, 76 winter, and one facultative) from Dagestan (Supplementary 1)1, among them the landraces (228 accessions) prevailed whereas only 36 accessions represented cultivars and breeding lines. The studied forms belong to the two subspecies: six-row barley (subsp. vulgare) and two-row barley (subsp. distichon) and represent 29 botanical varieties. Some accessions were registered in the collection of the N.I.Vavilov All-Russian Institute of Plant Genetic Resources (VIR) as populations involving up to five varieties. Fortyseven barley lines carrying previously identified powdery mildew resistance genes were also studied (Supplementary 2).


Supplementary materials 1–2 are available at: http://www.bionet.nsc.ru/vogis/download/pict-2021-25/appx10.pdf


Field trials were carried out at the Dagestan Experiment Station of VIR (DES VIR, Derbent; latitude 41°59ʹ03.3″N, longitude 48°19ʹ47.7″E) in 2012–2014. Accessions were sown in the third decade of October in the field plots of 1 m2 area, with a row to row spacing of 15 cm and row length of 1 m. Spring barley cultivar Temp (VIR-22055, Krasnodar region) sown after each 20 accessions was used as a control. Resistance to disease was evaluated at the heading stage and at the milk-ripe stage and expressed as infection type (IT) in the following scale (Loskutov et al., 2012):

IT 1 – resistance is very low – pustules cover all leaves and internodes in abundance, including the upper ones; a lesion can capture an ear;

IT 3 – low resistance – pustules in bulk develop mainly on the lower leaves and internodes; individual scattered pustules are observed on the upper leaves;

IT 5 – medium resistance – a moderate number of pustules on the lower leaves and internodes;

IT 7 – high resistance – single small pustules on the lower leaves and internodes, pustules can be more numerous, but small;

IT 9 – very high resistance – no pustules are visible.

To exclude the presence of known powdery mildew resistance genes in barley accessions from Dagestan, the seedling resistance test was performed. Fungal inoculum was propagated on plants grown in a Barnstead 845-2 climatic chamber at a 12-hour photoperiod and a temperature of 16 °C (day), 13 °C (night).

The Northwest (St. Petersburg, Pushkin) population of the fungus was used for inoculation. The population was collected from a susceptible barley cultivar Belogorsky (VIR-22089, Leningrad region). Twenty seeds of each of resistant accessions and 47 barley lines carrying previously identified powdery mildew resistance genes were sown on water-moistened cotton in plastic trays, placed in a climatic chamber, and after a week the seedlings were inoculated through shaking conidia on them from plants strongly affected by powdery mildew. Infection types were scored using 0–4 point scale (Mains, Dietz, 1930) as follows:

IT 0 – highly resistant, no mycelium evident. Chlorotic or necrotic spots may be developed by some varieties;

IT 1 – very resistant, slight to moderate mycelial development, but with little or no sporulation. Chlorotic or necrotic spots may develop in some varieties;

IT 2 – moderately resistant, moderate mycelial development, accompanied by limited sporulation. Chlorotic or necrotic areas may be formed;

IT 3 – moderately susceptible. Moderate to abundant mycelial development, accompanied by moderate sporulation;

IT 4 – very susceptible. Abundant mycelial development, accompanied by abundant sporulation.

To specify resistance genes, we have estimated segregation ratios in the F_2_ hybrid populations obtained from crossing resistant accessions with a susceptible variety (VIR-15033). For allelic testing, the resistant accessions were crossed with each other, as well as with near-isogenic line Ingrid *mlo11*. The resistance tests were conducted in a climatic chamber.

The germinated seeds were sown on water-moistened cotton in plastic trays. Each tray contained one row of each parental form (P_1_, P_2_) and F_1_ hybrid, and 7–8 rows of F_2_ plants. At the two-leaf stage, the seedlings were inoculated with the Northwest pathogen population (collected near St. Petersburg). Hybrids from the crossings of resistant accessions and a susceptible tester were evaluated for resistance at the time of death of susceptible parental form. The assessment of allelic relations among powdery mildew resistance genes was carried out at the time of death of susceptible control (cultivar Belogorsky) which was sown along with F2 plants in the same tray. Plants exhibiting infection type similar to that of either the susceptible parent or the control (IT scores of 3 or 4 according to the scale of E.B. Mains and S.M. Dietz (1930) were classified as homozygous susceptible (S). The resistant class (R) comprised plants similar in infection type to the resistant parental form (IT scores of 0–1).

For identifying the *mlo11* gene, the PCR markers (Table 1) developed by P. Piffanelli et al. (2004) were used. Total DNA was isolated from 7-day-old seedlings according to the method of D.B. Dorokhov and E. Kloke (1997). Amplification was carried out in a 25 μl volume reaction mixture containing 50–100 ng of genomic DNA, 1× reaction buffer, 2 mM MgCl_2_, 0.25 mM dNTP_s_, 0.25 μM of each primer, 1 U Taq DNA polymerase (Dialat Ltd). PCR was conducted on a MyCycler thermal cycler (Bio-Rad, USA). The protocol consisted of an initial cycle of denaturation at 94 °C for 5 min, followed by 35 cycles (94 °C for 30 s, 60 °C for 30 s and 72 °C for 1.5 min). Final extension was done at 72 °C for 10 min. Amplification products were analyzed by electrophoresis on 1.5 % agarose gels and visualized under ultraviolet light. Fragment size was estimated using FastRuler™ SM1113 DNA-marker (Fermentas).

**Table 1.**
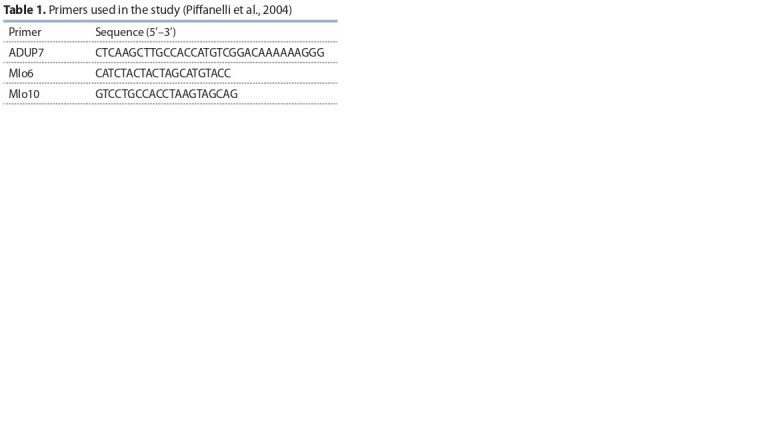
Primers used in the study (Piffanelli et al., 2004)

## Results

In 2012–2014, the epiphytotic disease development was observed at the DES VIR: the susceptible control cultivar Temp exhibited the IT score of 3 according to the 0–9 point scale (Loskutov et al., 2012). At a severe infection pressure, five accessions whose IT scores did not exceed 7 points have been initially isolated; in 2014 only two accessions (VIR-23787 and VIR-28212) were resistant (Table 2).

**Table 2.**
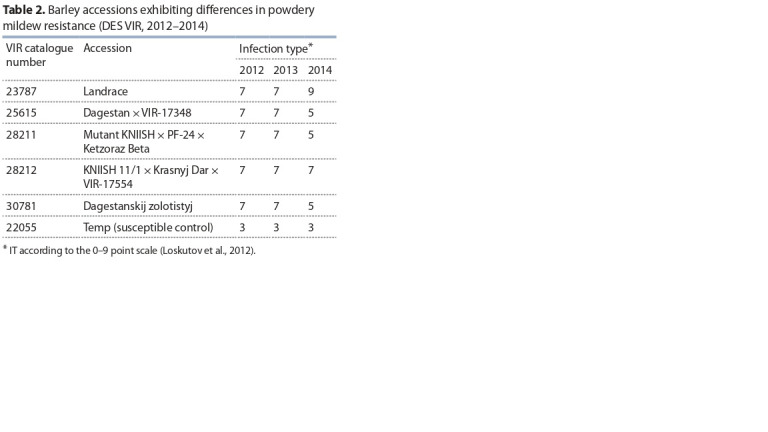
Barley accessions exhibiting differences in powdery mildew resistance (DES VIR, 2012–2014)

Forty-seven barley lines carrying previously identified powdery mildew resistance genes were evaluated under laboratory conditions and in the field experiments at the DES VIR. Twelve lines have been shown to be resistant to the pathogen in the field (Table 3). The same lines were resistant to the Northwest population of *B. graminis*, i. e. there were no significant differences in virulence between the two pathogen populations.

Two selected accessions, VIR-23787 and VIR-28212, exhibited resistance to the pathogen in the field and laboratory experiments. According to the information documented at the VIR Department of Oat, Rye and Barley Genetic Resources, the landrace accession VIR-23787 is of unknown pedigree, and line VIR-28212 originated from Ethiopian spring barley accession VIR-17554.

Thus, a comparative analysis of infection types of powdery mildew resistant accessions VIR-23787 and VIR-28212, and lines resistant to the Dagestan and St. Petersburg populations of *B. graminis* (see Table 3) has shown that these accessions might be protected either by the recessive gene(s) *mlo* or dominant *Mla*.

**Table 3.**
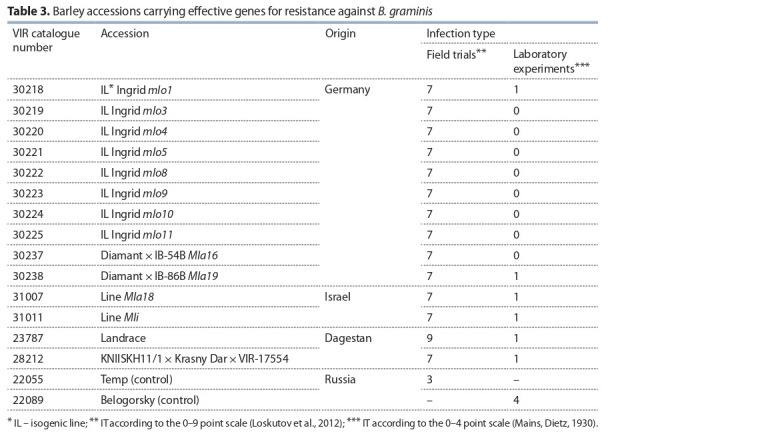
Barley accessions carrying effective genes for resistance against *B. graminis*

To determine the number of genes conferring resistance, the F_1_ and F_2_ hybrids obtained from crossing resistant accessions VIR-23787 and VIR-28212 with susceptible tester VIR-15033 were evaluated for resistance at the seedling stage. The parental genotypes VIR-23787 and VIR-28212 were resistant to the Northwest pathogen population with the IT score of 1, whereas F_1_ hybrid plants were susceptible to the pathogen (IT score of 4). Phenotypic segregation in the F_2_ populations fitted the expected ratio 1R: 3S (Table 4). We suggest that accessions VIR-23787 and VIR-28212 possess recessive genes conferring powdery mildew resistance at the seedling stage. Analysis of F_2_ hybrid progenies derived from reciprocal crosses between the resistant (VIR-28212) and susceptible (VIR-15033) accessions did not reveal an effect of the maternal or paternal genotype on the segregation pattern (χ ^2^_1:3_ = 0.60 and χ ^2^_1:3_ = 0.65).

**Table 4.**
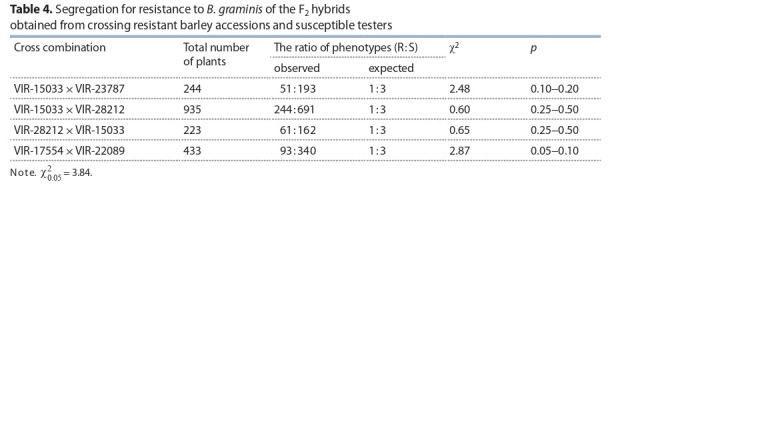
Segregation for resistance to *B. graminis* of the F2 hybrids obtained from crossing resistant barley accessions and susceptible testers

We have also analyzed segregation for pathogen resistance of the F_2_ hybrids obtained from crossing accession VIR-17554 from Ethiopia (a putative donor of disease resistance in the line VIR-28212) and susceptible cultivar Belogorsky (VIR-22089). The results indicated that accession VIR-17554 also carries a single recessive gene for *B. graminis* resistance (see Table 4).

The segregation pattern in the F_2_ generation allows us to suggest that *B. graminis* resistance of accessions VIR-28212 and VIR-17554 is controlled by the gene *mlo11*. There was no segregation in the F_2_ hybrid progenies from the crossings of these accessions with the *mlo11* allele carrier VIR-30225 (IL Ingrid *mlo11*) (Table 5). This means that accessions VIR-28212, VIR-17554 and VIR-30225 are protected by the *mlo11* gene.

**Table 5.**
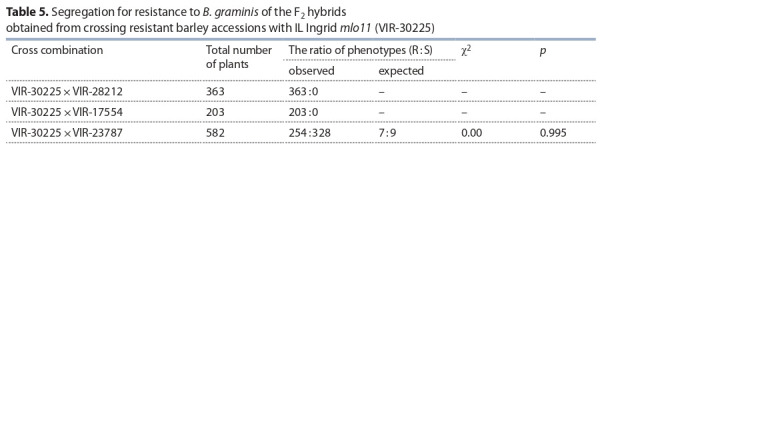
Segregation for resistance to B. graminis of the F2 hybrids obtained from crossing resistant barley accessions with IL Ingrid *mlo11* (VIR-30225)

Segregation in the F_2_ hybrid population from a cross between IL Ingrid *mlo11* and accession VIR-23787 fitted the expected ratio (7R: 9S; χ ^2^_7:9_ = 0.00) for two recessive resistance genes.

Accessions VIR-28212, VIR-17554 and VIR-23787 were analyzed with the use of the *mlo11* specific molecular markers (see Table 1). Amplification of specific 1200 and 440 bp fragments with primer pairs ADUP7-Mlo6 and Mlo6-Mlo10 respectively is usually an indication of the presence of the mlo11 gene. Genotypes carrying the *mlo11* allele were found in accessions VIR-17554 and VIR-28212 (see the Figure). No carriers of the *mlo11* allele were detected among the 20 plants analyzed in accession VIR-23787, therefore this form is protected by another resistance gene.

**Fig.**
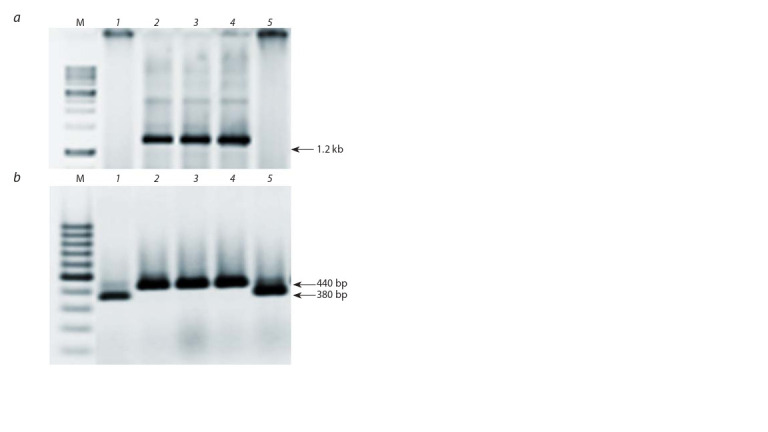
Molecular marker-based assessment of powdery mildew resistant barley plants with the use of primer pairs ADUP7-Mlo6 (a) and Mlo6-Mlo10 (b). a – the 1.2 kb fragment is specif ic for the *mlo11* allele; b – the 380 bp fragment is amplif ied in the genotypes with the wild-type *Mlo* allele and a fragment of approx. 440 bp size is amplified in the genotypes carrying the *mlo11* allele. 1 – susceptible control VIR-22089 (cultivar Belogorsky); 2 – line VIR-30225 with the *mlo11* allele; 3 – VIR-28212; 4 – VIR-17554; 5 – VIR-23787. M – molecular weight marker.

## Discussion

The results of the study indicate a rather low level of genetic diversity for powdery mildew resistance within the studied set of Dagestan barley accessions. Five accessions (VIR-23787, VIR-25615, VIR-28211, VIR-28212, VIR-30781) were selected for powdery mildew resistance in 2012 and 2013. However, in the past years their susceptibility is enhanced, which may be due to changes of the pathogen population structure. Only two accessions, VIR-23787 and VIR-28212, were resistant to the Dagestan population of *B. graminis* at the adult stage and to the St. Petersburg population of the pathogen at the seedling stage respectively. It is interesting that two geographically very distant populations of the fungus turned out to be identical in virulence to the tester lines (see Table 3).

It was somewhat surprising, that accession VIR-28212 was protected by the effective resistance gene *mlo11*, which was introgressed from VIR-17554 (Ep-80 Abyssinien). This accession, which has entered the VIR collection from the German Gene Bank in 1949, turned out to be heterogeneous for resistance (eight resistant plants out of the ten studied). Plants belonging to the variety nigrinudum are most likely a result of genetic contamination in consequence of the cross-pollination, which is quite typical for Ethiopian barley.

Durable non-specific resistance of barley to *B. graminis* is associated with mutations at the *Mlo* locus at the long arm of chromosome 4 (Jørgensen, 1992). The resistance of *mlo* mutants is associated with physiological processes which prevent successful penetration of the pathogen into epidermal cells of the host plant (Ge et al., 2016). Carriers of the recessive *mlo* allele are characterized by leaf damage which is considered as a manifestation of premature cell death symptoms after cell wall appositions (callose deposits on adult leaves), observed even in the absence of the pathogen (Skou et al., 1984). Despite a number of limitations associated with negative pleiotropic effects leading to yield decrease, the use of *mlo* alleles (mostly *mlo11* and, in part, *mlo9*) in barley breeding programs provided durable protection against *B. graminis* in the regions with temperate humid climate. Currently, 75 % of modern spring barley varieties in Europe are protected by the *mlo* genes (Dreiseitl, 2017).

The recessive *B. graminis* resistance gene in accession VIR-23787 is distinct from the *mlo11* and not associated with negative pleiotropic effects which are characteristic of the *mlo* alleles induced by chemical mutagenesis. In addition, unlike the accessions carrying recessive *mlo* alleles, no symptoms of fungal damage were detected on plants of accession VIR-23787. Therefore, we assume that this accession is protected by a new pathogen resistance gene.

Recently, a novel recessive gene conferring broad-spectrum resistance against *Blumeria graminis* f. sp. *hordei* was found in a spring barley line selected from a Moroccan landrace at the Polish Plant Breeding and Acclimatization Institute (Piechota et al., 2020). The gene designated mlmr was mapped at the long arm of chromosome 6H. In the other study performed with the use of phytopathological testing, a new resistance allele MlLu was identified among 16 winter barley accessions originating from four gene banks (Dreiseitl, 2019). Thus, identification of novel genes in barley landraces can facilitate the broadening of the available powdery mildew resistance germplasm. Moreover, knowledge of resistance phenotypes can assist in determining accessions authenticity and their genotype purity in gene banks (Dreiseitl, Zavřelová, 2018).

## Conclusion

The cultivation of barley varieties carrying effective genes for resistance against *B. graminis* f. sp. *hordei* can significantly limit the harmfulness of the pathogen. The specificity in the interaction between the fungus and its host plant requires a continuous search for new resistance donors. Barley landraces are traditionally considered as sources of novel genes for pathogen resistance. The present study performed under field and laboratory conditions has revealed rather low genetic diversity for effective resistance against powdery mildew within the examined set of 264 barley accessions from Dagestan. Only two accessions, VIR-23787 and VIR-28212, were resistant to the Dagestan population of *B. graminis* at the adult stage and to the St. Petersburg population of the pathogen at the seedling stage. Accession VIR-28212 is protected by the effective resistance gene *mlo11*, which was probably introgressed from accession VIR-17554 (Ethiopia). Accession VIR-23787 has another recessive *B. graminis* resistance gene which is distinct from *mlo11* and does not have negative pleiotropic effects typical for other *mlo* alleles obtained by chemical mutagenesis.

## Conflict of interest

The authors declare no conflict of interest.
